# Mitral Annular Disjunction Assessed Using CMR Imaging: Insights From the UK Biobank Population Study

**DOI:** 10.1016/j.jcmg.2022.07.015

**Published:** 2022-11

**Authors:** Dasa Zugwitz, Kenneth Fung, Nay Aung, Elisa Rauseo, Celeste McCracken, Jackie Cooper, Saloua El Messaoudi, Robert H. Anderson, Stefan K. Piechnik, Stefan Neubauer, Steffen E. Petersen, Robin Nijveldt

**Affiliations:** a Faculty of Medicine, University of Ljubljana, Ljubljana, Slovenia; b Department of Cardiology, Radboud University Medical Center, Nijmegen, the Netherlands; c William Harvey Research Institute, NIHR Barts Biomedical Research Centre, Queen Mary University of London, London, United Kingdom; d Barts Heart Centre, St Bartholomew’s Hospital, Barts Health NHS Trust, West Smithfield, London, United Kingdom; e Biosciences Institute, Newcastle University, Newcastle-upon-Tyne, United Kingdom; f Division of Cardiovascular Medicine, Radcliffe Department of Medicine, University of Oxford, Oxford, United Kingdom

**Keywords:** cardiac magnetic resonance, mitral annular disjunction, mitral valve prolapse, BMI, body mass index, CMR, cardiac magnetic resonance, CT, computed tomography, OR, odds ratio

## Abstract

**Background:**

Mitral annular disjunction is the atrial displacement of the mural mitral valve leaflet hinge point within the atrioventricular junction. Said to be associated with malignant ventricular arrhythmias and sudden death, its prevalence in the general population is not known.

**Objectives:**

The purpose of this study was to assess the frequency of occurrence and extent of mitral annular disjunction in a large population cohort.

**Methods:**

The authors assessed the cardiac magnetic resonance (CMR) images in 2,646 Caucasian subjects enrolled in the UK Biobank imaging study, measuring the length of disjunction at 4 points around the mitral annulus, assessing for presence of prolapse or billowing of the leaflets, and for curling motion of the inferolateral left ventricular wall.

**Results:**

From 2,607 included participants, the authors found disjunction in 1,990 (76%) cases, most commonly at the anterior and inferior ventricular wall. The authors found inferolateral disjunction, reported as clinically important, in 134 (5%) cases. Prolapse was more frequent in subjects with disjunction (odds ratio [OR]: 2.5; *P* = 0.02), with positive associations found between systolic curling and disjunction at any site (OR: 3.6; *P* < 0.01), and systolic curling and prolapse (OR: 71.9; *P* < 0.01).

**Conclusions:**

This large-scale study shows that disjunction is a common finding when using CMR. Disjunction at the inferolateral ventricular wall, however, was rare. The authors found associations between disjunction and both prolapse and billowing of the mural mitral valve leaflet. These findings support the notion that only extensive inferolateral disjunction, when found, warrants consideration of further investigation, but disjunction elsewhere in the annulus should be considered a normal finding.

So-called “mitral annular disjunction” is the separation between the left atrial wall, the hinge point of the mural mitral leaflet, and the base of the left ventricular free wall.^1^ First described in 1876,^2^ and systematically studied in the 1980s,^3^, ^4^, ^5^ the finding went largely unnoticed until recently, despite an early report suggesting it might be related to sudden cardiac death.^6^ The recent technical advances in echocardiography, and better accessibility of cardiac magnetic resonance (CMR), have now made it easier to observe this entity. In echocardiographic studies, disjunction has mostly been observed and described only adjacent to the inferolateral ventricular wall because this section is best visualized in the parasternal long axis view. A recent study by Dejgaard et al,^7^ however, reported on a detailed analysis using CMR in patients with suspected disjunction on echocardiography. They showed that disjunction was usually spread around a larger part of the annulus, being interspersed with normal hinging, concurring with previous histologic findings.^4^

A growing body of evidence has suggested that disjunction might play a role in arrhythmic events in patients with^8^, ^9^, ^10^, ^11^, ^12^ and without^7^ mitral valvar prolapse. Most of the published studies, however, have been conducted on preselected populations of patients.^10^ Thus far, retrospective studies have mostly been based on images obtained from consecutive patients referred for echocardiography.^13^^,^^14^ A recent study, nonetheless, observed disjunction with computed tomography (CT) in structurally normal hearts.^15^ There is, however, a paucity of data on the prevalence and circumferential extent of disjunction in the general population. Indeed, to our knowledge, there have been no studies on disjunction in subjects without clinical indications for CMR. Our aim, therefore, was to assess the prevalence and extent of disjunction in a large cohort with no clinical indication for CMR. Additionally, we aimed to seek any association between disjunction and prolapse or incident arrhythmias. Such information is essential if we are better to understand and refine approaches to the diagnosis of this feature, and its risk stratification.

## Methods

### Study population

In this observational cross-sectional study, we analyzed the CMR images from 2,646 Caucasian subjects enrolled between April 2014, and August 2015, in the UK Biobank imaging study.^16^ The selection included 804 subjects without any known cardiovascular disease, other serious illnesses, or risk factors for cardiovascular disease, who have previously been selected for the study, which provided the specific reference ranges for chamber quantification.^17^ Of these, 35 were later diagnosed with either cardiovascular disease or other illnesses and removed from the healthy cohort. We then made a random selection of 1,842 scans from the remaining 4,261 scans available in the UK Biobank database, which were obtained within the selected time period (Supplemental Figure 1). Although these participants did not fit the strict criteria used in the aforementioned study, it has been shown that the participants are, in general, healthier, leaner, and with lower rate of all-cause mortality and lower total cancer incidence than the UK population taken as a whole.^18^ Analysis of the health outcomes for these participants is further described in Results. This study complies with the Declaration of Helsinki; the work was covered by the ethical approval for UK Biobank studies from the National Health Service National Research Ethics Service on June 17, 2011 (Ref 11/NW/0382) and extended on June 18, 2021 (Ref 21/NW/0157) with written informed consent obtained from all participants.

### Imaging

The full CMR protocol used in the UK Biobank has been described in detail elsewhere.^16^ In short, all examinations were performed on a clinical wide-bore 1.5-T scanner (MAGNETOM Aera, Syngo Platform VD13A, Siemens Healthcare). All acquisitions used a balanced steady-state free precession cine sequence with the following typical parameters: TR/TE = 2.7/1.2 ms, flip angle 80°, Grappa factor 2, voxel size 1.8 mm × 1.8 mm × 6 mm, and acquired temporal resolution 32.64 ms.

### Image analysis

**Figure 1:**
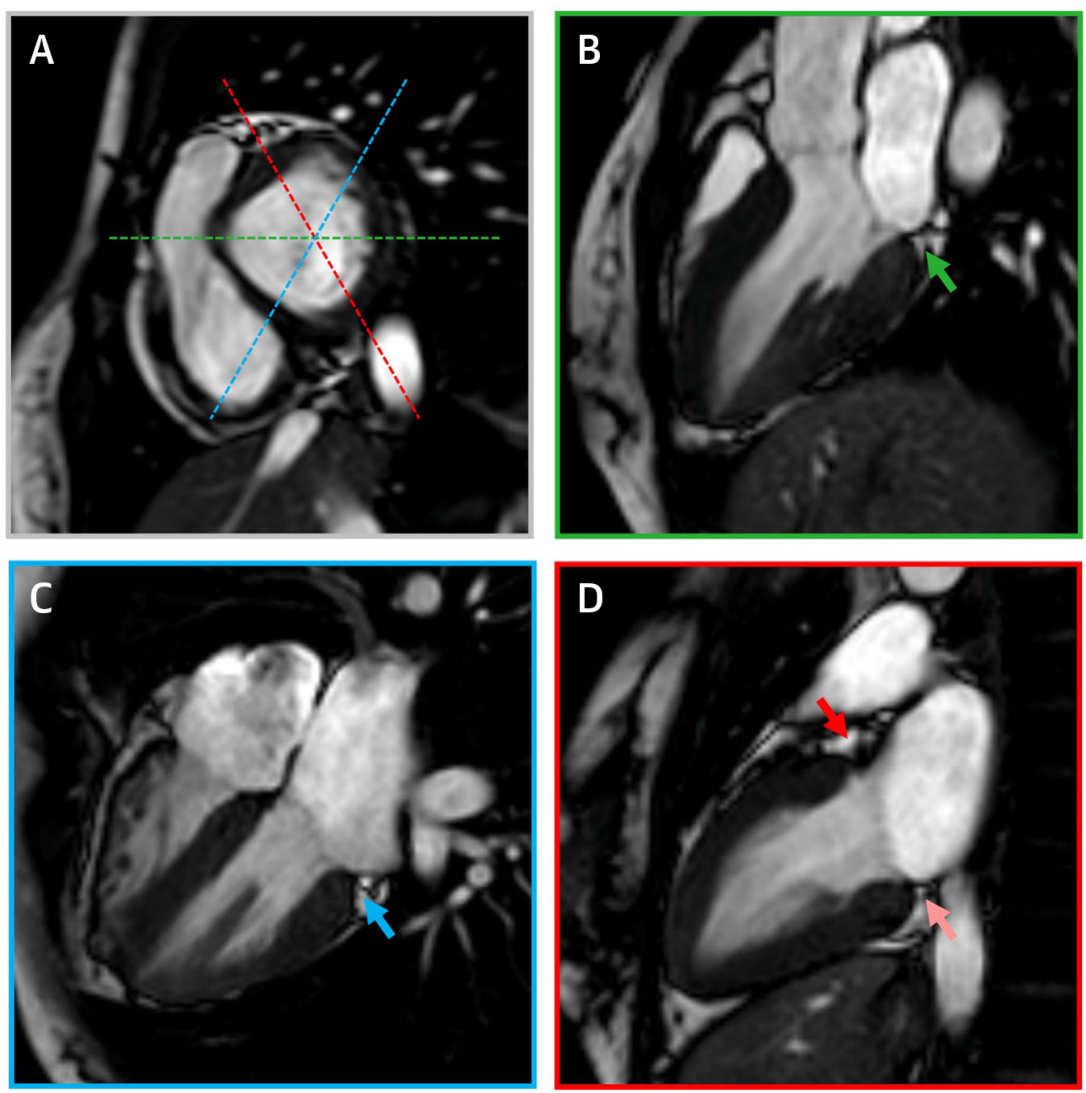
CMR Protocol Cardiac magnetic resonance (CMR) short-axis view of the atrioventricular valve plane **(A)** and long-axis views **(B to D)** displaying the planning protocols with corresponding imaging planes used for assessment of mitral atrioventricular disjunction. **Color-coded dashed lines** in the short-axis view correspond with corresponding color-framed long-axis views. **Arrows** point to the site of atrioventricular junction assessed for disjunction: inferolateral **(green arrow)**, inferior **(light red arrow)**, anterior **(dark red arrow)**, anterolateral **(blue arrow)**.

**Figure 2:**
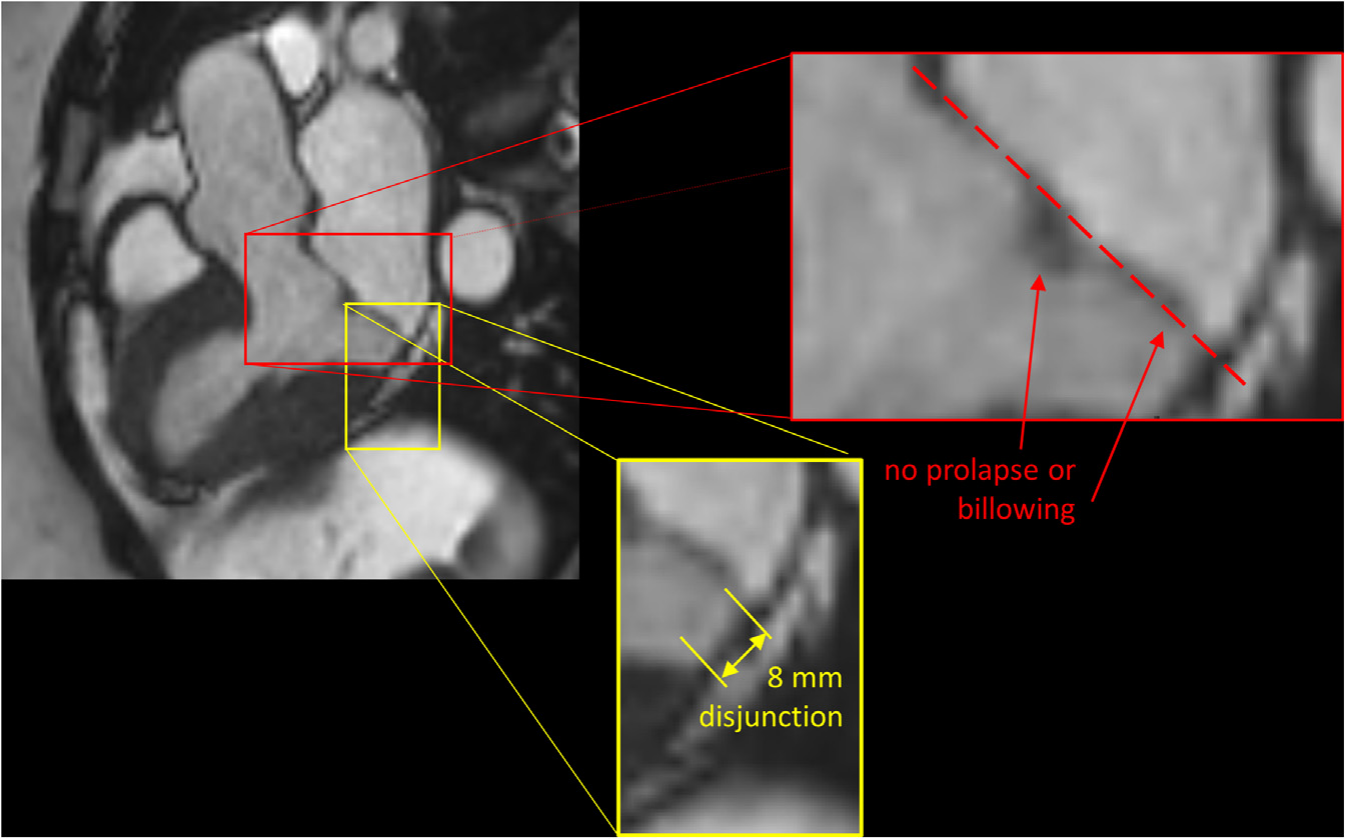
CMR in a Patient With Inferolateral Disjunction Without Mitral Valve Prolapse or Billowing CMR 3-chamber long-axis view **(upper left)** with enlarged details displaying measurement of inferolateral disjunction **(yellow frame)** and absence of mitral valve prolapse or leaflet billowing **(red frame)** as assessed by drawing a line in the virtual annular plane **(red interrupted line)**. © UK Biobank, by kind permission. Abbreviation as in Figure 1.

A standard operating procedure for analysis of disjunction was developed and agreed to before study commencement. Scans were analyzed using cvi42 postprocessing software version 5.10 (Circle Cardiovascular Imaging Inc). The images were first assessed for quality of long-axis views and presence of artifacts. In scans with sufficient quality, the long-axis cine images were visually analyzed for the presence of disjunction at the attachment of mural, or posterior, leaflet of the mitral valve to the anterior, anterolateral, inferolateral, and inferior segments of the annulus using standardized myocardial segmentation nomenclature (Figure 1).^19^ In this regard, it should be noted that, if considered attitudinally, the segment said to be “anterior” would better be described as being “superior,” whereas the “anterolateral” segment is posteriorly located when assessed relative to the bodily coordinates. We have retained, nonetheless, these conventional descriptors when denoting the site of measured disjunction. Disjunction was defined as present when it measured 1 mm or more, observing the consensus statement for CMR.^20^ Where disjunction was observed, it was measured from the top edge of the ventricular wall to the hinge of the leaflet from the left atrial wall, parallel to disjunction at end-systole (Figure 2). The end-systolic phase was selected by determining the phase in which the intracavity ventricular blood pool was at its smallest. The 3-chamber view was assessed for the presence of either prolapse or billowing of either leaflet of the mitral valve (Figure 2). Prolapse was classified as systolic displacement of any part of the leaflet by 2 mm or more from the annular plane into the left atrium in 3-chamber view as recommended by the American Society of Echocardiography,^21^ the more common standard criterion used for definition of prolapse in the clinical setting. Billowing was defined as systolic protrusion of the leaflet of <2 mm above the junctional plane with the coaptation point at or below the plane at end-systole, thus capturing cases of overabundant leaflet tissue not reaching the criteria for prolapse. The images were also assessed for so-called systolic curling, a feature represented by excessive end-systolic motion of the inferolateral hinge point of the mural leaflet relative to the ventricular mural summit, and previously related to disjunction in patients with arrhythmic prolapse.^22^ Ventricular volumes and ejection fraction were measured using manual tracing as described elsewhere.^17^

### Interobserver and intraobserver quality assessment

Image analysis was performed, according to the standard operating procedure, by an experienced physician with training in magnetic resonance imaging (D.Z.), and supervised by an experienced cardiologist with Level 3 certification in CMR as assessed by the European Association for Cardiovascular Imaging. Interobserver and intraobserver variability was assessed by analyzing 100 randomly selected scans, which were reassessed by D.Z. after a 1-month interval, and assessed by another physician with extensive training (E.R.).

### Clinical data

Sex at birth was recorded at the baseline visit. Age, body mass index (BMI), and blood pressure were recorded at the time of imaging. Clinical diagnoses including arrhythmic events were derived using a combination of UK Biobank fields, including self-reported illness, medication use, inpatient diagnoses, and algorithmically derived outcomes.^23^ A detailed description of fields and values is given in Supplemental Table 1.

### Statistical analysis

Statistical analysis was performed using R version 4.0.3^24^ and R Studio version 1.3.1093.^25^ Continuous variables were presented as mean ± SD and categorical data as number (percentage). Group-wise comparisons were performed using Student *t*-test, Fisher exact test, Kruskal-Wallis test, and 1-way analysis of variance. Fisher exact tests were used to test for relationships between disjunction status and other features. Multivariable logistic modelling was used to explore these relationships further, adjusting for age, sex, arterial hypertension, and BMI. Correlation analyses were made using Pearson correlations. Two-sided values of *P* ˂ 0.05 were considered significant. Intraobserver and interobserver measurement reliability was assessed using intraclass correlation coefficient.

## Results

### Study population

We analyzed visually a total of 2,646 scans. Of these, 39 were discarded due to insufficient quality of any of the long-axis views, retaining the remaining 2,607 datasets (Supplemental Figure 1). Of these, 1,383 (52%) were from females. The average age of the participants was 61.3 ± 7.5 years, measuring 170 ± 9 cm, weighing 75 ± 15 kg, and with BMI of 25.9 ± 4.3 kg/m^2^ (Table 1). Of the selected population, 747 subjects have reported or have been diagnosed with arterial hypertension, 134 had diagnosed ischemic heart disease, and just 5 subjects had any other cardiomyopathy in the UK Biobank database. Only 68 subjects had either reported or had been diagnosed with cardiac arrhythmias of any kind (Supplemental Table 1). Of these, just 6 were ventricular arrhythmias, and 4 people survived cardiac arrest for any cause before imaging.

**Table 1:** Baseline Characteristics

	Overall (N = 2,607)	Healthy Cohort (n = 769)	Subjects With CVD or Risk Factors (n = 1,016)	Subjects With Non-CVD Morbidity (n = 822)	*P* Value	Test
Clinical characteristics						
Female	1,358 (52.1)	416 (54.1)	423 (41.6)	519 (63.1)	<0.001	1
Age, y	61.3 ± 7.5	59.1 ± 7.1	64.2 ± 6.9	59.8 ± 7.5	<0.001	2
Height, cm	170.1 ± 9.3	170.4 ± 9.2	170.7 ± 9.5	169.2 ± 9.2	0.001	2
Weight, kg	75.1 ± 15.1	69.7 ± 12.0	79.7 ± 15.6	74.5 ± 15.3	<0.001	3
BMI, kg/m^2^	25.9 ± 4.3	23.9 ± 2.8	27.3 ± 4.6	25.9 ± 4.5	<0.001	3
DBP, mm Hg	78.6 ± 9.7	76.7 ± 9.0	80.8 ± 9.9	77.6 ± 9.6	<0.001	2
SBP, mm Hg	136.6 ± 17.8	131.4 ± 16.3	143.1 ± 17.6	133.4 ± 17.1	<0.001	2
CMR variables						
LVEDV, mL	144.7 ± 34.3	143.7 ± 34.1	147.1 ± 35.3	142.7 ± 33.1	0.021	3
LVEF, %	59.6 ± 6.4	59.5 ± 5.7	59.4 ± 6.9	59.8 ± 6.3	0.328	2
LVESV, mL	59.2 ± 19.9	58.5 ± 17.6	60.7 ± 21.8	58.0 ±19.3	0.098	3
LVM, g	88.8 ± 24.7	85.3 ± 23.7	94.6 ± 25.3	84.8 ± 23.5	<0.001	3
LVSV, mL	85.5 ± 19.4	85.1 ± 20.3	86.4 ± 19.1	84.7 ± 19.0	0.025	3
RVEDV, mL	154.0 ± 38.3	154.5 ± 40.2	155.5 ± 37.6	151.8 ± 37.1	0.083	3
RVEF, %	56.4 ± 6.7	55.9 ± 6.3	56.5 ± 6.9	56.6 ± 6.8	0.075	2
RVESV, mL	68.2 ± 23.2	69.2 ± 23.8	68.5 ± 23.1	66.9 ± 22.6	0.217	3
RVSV, mL	85.8 ± 19.7	85.4 ± 20.3	87.0 ± 19.5	84.9 ± 19.2	0.017	3
Disjunction occurrence and length						
Disjunction present any site	1,990 (76.3)	617 (80.2)	730 (71.9)	643 (78.2)	<0.001	1
Anterolateral, present	329 (12.6)	122 (15.9)	89 (8.8)	118 (14.4)	<0.001	1
Anterolateral, mm, when present	2.7 ± 1.0	2.6 ± 1.0	2.9 ± 1.2	2.7 ± 0.9	0.18	3
Anterior, present	1,413 (54.2)	436 (56.7)	530 (52.2)	447 (54.4)	0.209	1
Anterior, mm, when present	2.6 ± 0.9	2.6 ± 0.9	2.7 ± 1.0	2.6 ± 0.9	0.549	3
Inferior, present	1,522 (58.4)	474 (61.6)	555 (54.6)	493 (60.0)	0.013	1
Inferior, mm, when present	3.4 ± 1.4	3.4 ± 1.4	3.5 ± 1.4	3.3 ± 1.3)	0.085	3
Inferolateral, present	134 (5.1)	48 (6.2)	41 (4.0)	45 (5.5)	0.101	1
Inferolateral, mm, when present	3.2 ± 1.3	3.1 ± 1.1	3.1 ± 1.5	3.3 ± 1.3	0.474	3
Prolapse, curling, and billowing						
Prolapse	76 (2.9)	26 (3.4)	24 (2.4)	26 (3.2)	0.392	1
Prolapse anterior leaflet	6 (0.2)	2 (0.3)	2 (0.2)	2 (0.2)	1.000	1
Prolapse posterior leaflet	61 (2.3)	19 (2.5)	20 (2.0)	22 (2.7)	0.589	1
Prolapse bileaflet	9 (0.3)	5 (0.7)	2 (0.2)	2 (0.2)	0.278	1
Inferolateral curling	51 (2.0)	23 (3.0)	9 (0.9)	19 (2.3)	0.003	1
Posterior leaflet billowing	34 (1.3)	12 (1.6)	10 (1.0)	12 (1.5)	0.515	1

Values are mean ± SD or n (%). Test 1 = Fisher exact test for count data; Test 2 = F-test from 1-way analysis of variance; Test 3 = Kruskal-Wallis nonparametric 1-way analysis of variance. Kruskal-Wallis was applied where the average absolute group skewness was >0.5.

BMI = body mass index; CMR = cardiac magnetic resonance; CVD = cardiovascular disease; DBP = diastolic blood pressure; LVEDV = left ventricular end-diastolic volume; LVEF = left ventricular ejection fraction; LVESV = left ventricular end-systolic volume; LVM = left ventricular mass; LVSV = left ventricular stroke volume; RVEDV = right ventricular end-diastolic volume; RVEF = right ventricular ejection fraction; RVESV = right ventricular end-systolic volume; RVSV = right ventricular stroke volume; SBP = systolic blood pressure.

### Disjunction

Disjunction was found in at least 1 of the chosen sites in 1,990 cases (76%), being found inferiorly in 58% of cases, anteriorly in 54%, and anterolaterally in 13%. Inferolateral disjunction was found in only 5%. The extent of disjunction varied significantly between the sites, with the longest segment, of 1 cm, found inferiorly. The longest average disjunction, if present, at 3.4 ± 1.4 mm, was also found inferiorly. Average lengths, when present, anteriorly, anterolaterally, and inferolaterally were 2.6 ± 0.9 mm, 2.7 ± 1.0 mm, and 3.2 ± 1.3 mm, respectively (Table 1). Significant, albeit weak to moderate, positive correlations of co-occurrence were found between sites, with the strongest correlations found for the rarest co-occurrence, which was inferolateral and anterolateral (*r* = 0.427; *P* < 0.001). We found 3 dominant patterns, accounting for 85% of studied subjects. A single site of disjunction, either inferiorly or anteriorly, was found in 32%, with 30% having inferior and anterior disjunction and 24% having no disjunction (Table 2).

**Table 2:** Prevalence and Patterns of Occurrence of Annular Disjunction

No. of Site(s) of Observed Disjunction	Distribution Pattern(s) of Disjunction When Observed	Participants
0: 620 (24%)	None	620 (23.8)
1: 865 (33%)	Inferior	451 (17.3)
	Anterior	380 (14.6)
	Anterolateral	30 (1.2)
	Inferolateral	4 (0.2)
2: 886 (34%)	Inferior and anterior	774 (29.7)
	Inferior and anterolateral	56 (2.1)
	Anterior and anterolateral	40 (1.5)
	Inferior and inferolateral	13 (0.5)
	Anterolateral and inferolateral	2 (0.1)
	Anterior and inferolateral	1 (0.0)
3: 183 (7%)	Inferior, anterior, anterolateral	122 (4.7)
	Inferior, anterior, inferolateral	35 (1.3)
	Inferior, anterolateral, inferolateral	18 (0.7)
	Anterior, anterolateral, inferolateral	8 (0.3)
4: 53 (2%)	All sites	53 (2.0)

Values are n (%).

### Disjunction and disease

No statistically significant differences were found between groups when comparing the length of disjunction between healthy individuals and those with either at least 1 known risk factor for disease or overt disease (Table 1). After adjusting for age, sex, and BMI, we found no significant association between disjunction length and any cardiac arrhythmia. With ventricular arrhythmias reported in only 6 individuals, no further analysis could be done. Due to the low number of individuals with known disease, no analysis could be done to assess associations with heart failure, valvar heart disease, or nonischemic cardiomyopathies.

### Disjunction, prolapse, and billowing

These features, involving either leaflet of the mitral valve, were present in 109 cases (4.1%), with 33 individuals exhibiting billowing. Prolapse of the aortic leaflet and billowing of the mural leaflet was found in 1 case. Prolapse was found in 76 (3.0%) individuals, with 6 having prolapse of the aortic leaflet, 61 of the mural leaflet, and 9 of both leaflets.

We found a strong association between the disjunction and a general increased prevalence of prolapse (Table 3), particularly of the mural leaflet (Table 4). Billowing of the mural leaflet, indicating abundant leaflet tissue without prolapse, was also associated with an increased prevalence of disjunction (Table 4).

**Table 3:** Prevalence of Prolapse in Association With Disjunction

Disjunction Variable		Outcome Variable Prolapse	Fisher Test^a^*P* Value	Logistic Regression^b^		
				Odds Ratio	*P* Value	Model
Disjunction present at any site	Present	68/1,974 (3.4)	0.0056	2.5 (1.3-5.7)	0.0161	1. Single exposure
	Absent	8/606 (1.3)				
Inferolateral	Present	13/134 (9.7)	1.03 x 10^-4^	2.2 (1.0-4.4)	0.0417	2. Simultaneous exposures
	Absent	63/2,445 (2.6)				
Inferior	Present	61/1,508 (4.0)	1.22 x 10^-4^	2.3 (1.3-4.2)	0.0071	
	Absent	15/1,028 (1.5)				
Anterior	Present	50/1,405 (3.6)	0.0786	1.3 (0.8-2.1)	0.3270	
	Absent	26/1,121 (2.3)				
Anterolateral	Present	18/325 (5.5)	0.0070	1.2 (0.6-2.2)	0.5362	
	Absent	57/2,234 (2.6)				

Values are n/N (%) unless otherwise indicated.

There are 2 logistic models represented, one for the odds of prolapse given any disjunction overall, and another model including disjunction indicators across the 4 sites simultaneously.

a Fisher exact test for independence between 2 categorical variables. In each case, this is between prolapse (present/absent) and the disjunction variable listed (present/absent).

b Logistic models have prolapse as the outcome (present/absent), disjunction as the exposure, and are adjusted by age, sex, arterial hypertension, and body mass index.

**Table 4:** Prevalence of Mural Leaflet Prolapse and/or Billowing in Association With Disjunction

Disjunction Variable		Outcome Variable Mural Leaflet Prolapse	Fisher Test^a^*P* Value	Logistic Regression^b^	
				Odds Ratio	*P* Value
Inferolateral	Present	11/134 (8.2)	7.65 x 10^-4^	2.3 (1.1-4.5)	0.0184
	Absent	59/2,445 (2.4)			
Inferior	Present	58/1,508 (3.8)	3.32 x 10^-5^	2.9 (1.6-5.8)	0.0010
	Absent	12/1,028 (1.2)			

Disjunction Variable		Prolapse of Either Leaflet or Billowing	*P* Value	Odds Ratio	*P* Value
Inferolateral	Present	20/134 (14.9)	1.60 x 10^-7^	3.2 (1.8-5.4)	3.27 x 10^-5^
	Absent	84/2,445 (3.4)			
Inferior	Present	87/1,508 (5.8)	1.07 x 10^-7^	3.1 (1.9-5.6)	3.11 x 10^-5^
	Absent	17/1,028 (1.7)			

Values are n/N (%), unless otherwise indicated.

a Fisher exact test for independence between 2 categorical variables. In each case, this is between the outcome variable listed (present/absent) and the disjunction variable listed (present/absent).

b There are 2 logistic models represented, one for each outcome variable listed, and both have inferolateral and inferior disjunction indicator variables entered together as exposures. Logistic models are adjusted by age, sex, arterial hypertension, and body mass index.

**Figure 3:**
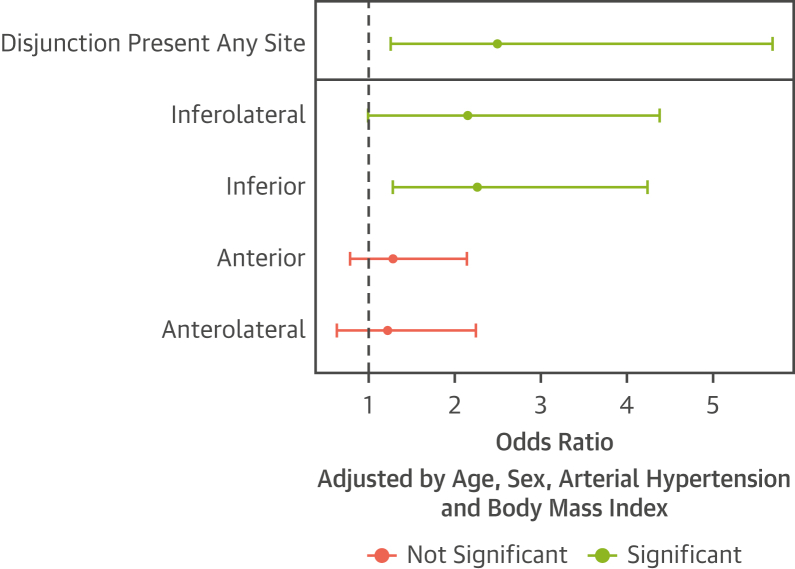
Odds Ratio for Mitral Valve Prolapse in Subjects With Disjunction Adjusted odds ratio of mitral valve prolapse in subjects with disjunction, cumulative and by site. There are 2 logistic models represented, one for the odds of prolapse given any disjunction overall, and another model including disjunction indicators across the 4 sites simultaneously. Both models are adjusted by age, sex, arterial hypertension, and body mass index.

Presence of disjunction at any site increased the odds of prolapse by >2-fold (odds ratio [OR]: 2.5). with inferolateral and inferior disjunction specifically associated with an increased prevalence (OR: 2.2 and OR: 2.3, respectively) (Table 3, Figure 3), but not anterior and anterolateral disjunction.

Disjunction can only be present at the insertion point of the mural leaflet. Hence, we further analyzed our data regarding disjunction and prolapse by excluding cases with isolated prolapse of the aortic leaflet. Focusing solely on inferolateral and inferior disjunction and excessive excursion of the mural leaflet either in terms of billowing or prolapse, we found a statistically significant correlation, with ORs of >2 for all (Table 4).

### Disjunction and systolic curling

Systolic curling was found in 51 participants, 71% of whom were female. In 66.7% of these, there was either prolapse or disjunction, but not both. In 7 individuals (14%), systolic curling was present in the absence of either prolapse or adjacent disjunction. The length of inferolateral disjunction, where present, was 1.8-6.5 mm, with a mean of 3.3 ± 1.2 mm.

Presence of disjunction was associated with systolic curling (OR: 3.6), most strongly when inferolateral (OR: 12.0; Table 5). Systolic curling was also strongly associated with prolapse in general, and mural prolapse in particular (OR: 71.9 and OR: 69.7, respectively), as well as billowing (OR: 10.3).

**Table 5:** Prevalence of Systolic Curling in Relation With Disjunction, Prolapse, or Billowing

		Outcome Variable	Fisher Test^a^	Logistic Regression^b^		
Exposure Variable		Systolic Curling	*P* Value	Odds Ratio	*P* Value	Model
Disjunction present any site	Present	47/1,974 (2.4)	0.0067	3.6 (1.5-12.1)	0.0144	1. Curling by disjunction
	Absent	4/605 (0.7)				
Inferolateral	Present	24/134 (17.9)	1.36 x 10^-18^	12.0 (5.9-24.5)	8.94 x 10^-12^	2. Curling by disjunction (4 sites simultaneously)
	Absent	27/2,445 (1.1)				
Inferior	Present	44/1,508 (2.9)	3.79 x 10^-5^	2.7 (1.2-6.8)	0.0238	
	Absent	7/1,028 (0.7)				
Anterior	Present	32/1,405 (2.3)	0.3221	0.9 (0.5-1.7)	0.7671	
	Absent	19/1,121 (1.7)				
Anterolateral	Present	21/325 (6.5)	2.55 x 10^-7^	1.2 (0.6-2.5)	0.6069	
	Absent	30/2,233 (1.3)				
Prolapse	Present	30/76 (39.5)	1.45 x 10^-35^	71.9 (37.1-143.0)	9.48 x 10^-36^	3. Curling by prolapse
	Absent	21/2,503 (0.8)				
Prolapse of mural leaflet	Present	28/70 (40.0)	4.05 x 10^-33^	69.7 (35.5-140.1)	4.27 x 10^-34^	4. Curling by mural leaflet prolapse
	Absent	23/2,509 (0.9)				

Values are n/N (%) unless otherwise indicated.

a Fisher exact test for independence between 2 categorical variables. In each case, this is between systolic curling (present/absent) and the exposure variable listed (present/absent).

b There are 4 logistic models represented, each with systolic curling as the outcome (present/absent), and the exposure variables as listed. Logistic models are adjusted by age, sex, arterial hypertension, and body mass index.

### Interobserver and intraobserver reliability

Intraobserver reliability was excellent, with intraclass correlation coefficient ≥0.93 for all sites measured. Interobserver measurement reliability was good to excellent, with the lowest reliability for the inferolateral site, with intraclass correlation coefficient of 0.72 (Supplemental Table 2, Supplemental Figure 2).

## Discussion

Our study reveals new insights into the feature described as mitral annular disjunction, adding to overall understanding of the entity, and its occurrence in a large population. As far as we are aware, ours is the largest study to date, and the first to examine its presence, extent, and size in a group of individuals without any clinical indication for CMR, and with no preselection bias. Our most important finding is the frequency of the finding in individuals without either disease of the mitral valve or any history of arrhythmia.

### Origin and frequency of disjunction

We observed the feature in at least 1 site in 76% of our analyzed scans, although prevalence between sites differed (Central Illustration). It was found most frequently inferiorly and anteriorly (ie, superior). This kind of bimodal distribution has recently been described in a CT analysis of structurally normal hearts.^15^ This finding also aligns well with the CMR study by Dejgaard et al.^7^ Anatomically speaking, the mitral annulus is a fibrous sheet-like, or band-like, structure within the mural atrioventricular junction. It is not a continuous ring or cord extending throughout the mural junction, which itself extends between the fibrous trigones anchoring the aortic-mitral unit within the base of the ventricular cone.^26^^,^^27^ To a degree, our findings reconcile the opposing views on the nature and commonness of disjunction from 2 early descriptions.^3^^,^^5^ Disjunction anywhere around the mural leaflet is a common finding, as observed by Angelini et al.^4^ Inferolateral disjunction, however, adjacent to the P2 scallop of mural leaflet, is fairly rare, as noted by Hutchins et al.^3^ Our observed prevalence, at 5%, was similar to their finding of 4.6%.^3^

**Central Illustration:**
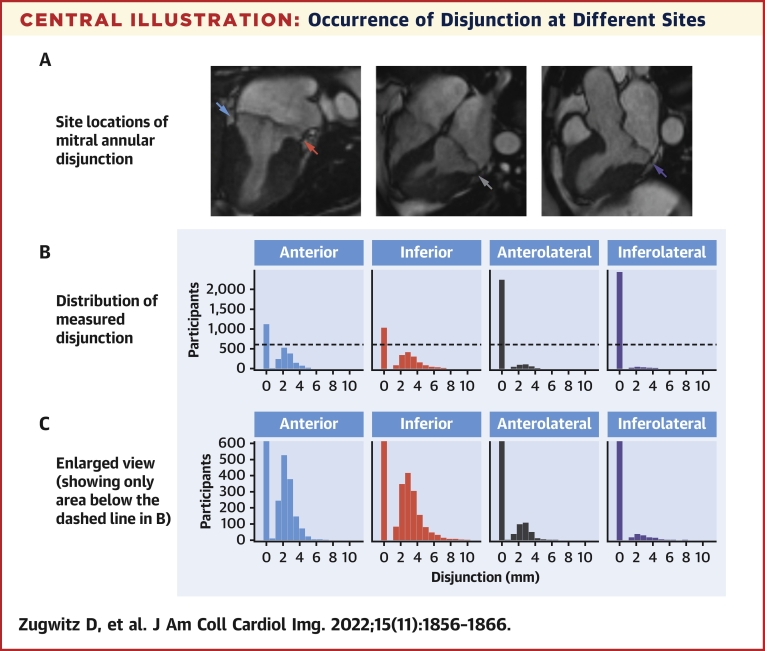
Occurrence of Disjunction at Different Sites CMR long-axis views with **arrows** pointing to the site of atrioventricular junction assessed for disjunction: inferolateral **(purple arrow)**, inferior **(red arrow)**, anterior **(blue arrow)**, and anterolateral **(grey arrow) (A)**; corresponding color-coded density graphs showing distribution of disjunction with left-handed bar denoting 0 disjunction **(B)** with enlarged view for clarification **(C)**. © UK Biobank, by kind permission.

### Disjunction, prolapse, and systolic curling

Both inferolateral disjunction and systolic curling of the mural leaflet relative to the basal segment of the inferolateral ventricular wall have been associated with hypermobility of the atrioventricular junction. Such a process believably causes mechanical injury of the adjacent ventricular myocardium via increased myocardial stretch, thus causing the replacement fibrosis seen as late gadolinium enhancement on CMR.^22^ It is believed that the excess force exerted on the weakened myocardium leads to arrhythmias,^28^ and that disjunction itself, rather than prolapse, causes the excessive mobility.^22^ Although our data confirms the association between inferolateral disjunction and systolic curling, we found a much stronger association of curling with prolapse rather than inferolateral disjunction (OR: 71.9 vs OR: 12.0). A considerable number of subjects with systolic curling (14%), nonetheless, had neither inferolateral disjunction nor prolapse, suggesting that the finding is not pathognomonic and other insofar unknown factors play a role in systolic curling.

### Clinical implications

Our results suggest that disjunction is a far more common finding in the general population than previously thought. So-called inferolateral disjunction, specifically in the part of the junction supporting the P2 scallop, was found in only 5%. Such disjunction is a common finding in patients with prolapse,^22^^,^^29^ myxomatous mitral valvar disease,^1^^,^^3^^,^^30^ and other connective tissue diseases.^31^ Our data corroborates the association between disjunction and prolapse. Importantly, it adds to the growing understanding of the nature of the feature by showing that this holds true not only for inferolateral, but also for inferior disjunction. But, because we found inferior disjunction in 58% of our study population, its clinical implications are questionable. Our findings challenge the premise that disjunction in itself, or even isolated inferolateral disjunction, is a risk marker for arrhythmic events, with only 6 reported cases of ventricular arrhythmia in the whole studied population, of which 1,990 have disjunction. A recently published study in patients with connective tissue diseases found an unusually high prevalence of disjunction. Despite reports of large inferolateral disjunction, there were few recorded ventricular arrhythmias.^31^ Much has yet to be learned about disjunction and its role in mechanical and electrical disturbance of cardiac function. There is evidence of a possible genetic cause for arrhythmic bileaflet prolapse.^32^ This might explain why disjunction is more common in individuals with prolapse of both leaflets,^28^ even in the absence of any direct anatomical relationship between disjunction and the aortic leaflet of the mitral valve.

### Study limitations

Our retrospective study had a cross-sectional design. The average age of our subjects when scanned was 61 years, whereas the median reported age for sudden cardiac death in patients with prolapse is 30 years.^33^ Our cohort, therefore, represents subjects at lower risk for sudden death due either to prolapse or disjunction. At the same time, our observed prevalence of disjunction suggests a more benign connotation for asymptomatic disjunction.

Late gadolinium enhancement images were unavailable, so we were unable to analyze if, and to what extent, isolated disjunction in asymptomatic individuals was related to scarring of either the papillary muscle or the inferolateral wall, as described in patients with arrhythmic prolapse.^22^^,^^34^ Additionally, there is paucity of data on normal mitral annulus dimension for different subgroups of people with regard to body surface area and other factors. A larger, multicenter registry-type study may be justified to investigate the clinical implications of inferolateral disjunction, especially in combination with prolapse and systolic curling in other age groups and ethnicities.

## Conclusions

Our results show that disjunction, as revealed by CMR, is by no means rare. Inferolateral disjunction, however, is infrequent. The shown prevalence and bimodal distribution of disjunction should further improve our understanding of the normal atrioventricular junction and prevent overdiagnosis of pathologic disjunction in healthy individuals.

Perspectives **COMPETENCY IN MEDICAL KNOWLEDGE:** There is limited understanding of the clinical significance of mitral annular disjunction, which is reflected in the unstandardized approach to its reporting. Presence of disjunction in 76% of study subjects, with bimodal distribution around the mitral valve annulus, should warrant against reporting of the finding as pathologic in the absence of other imaging and clinical criteria suggesting its clinical relevance.

**TRANSLATIONAL OUTLOOK:** Further imaging studies are needed to determine the length of normal annulus from pathologic disjunction, which will lead to better guidelines on the reporting of disjunction. Additional clinical studies, especially prospective studies in young individuals with complex ventricular arrhythmias, are needed to assess the true role and clinical significance of disjunction.

## Funding Support and Author Disclosures

This work was partly funded by the European Union’s Horizon 2020 research and innovation program under grant agreement number 825903 (euCanSHare project, Dr Petersen). Dr Petersen acknowledges support from the National Institute for Health Research (NIHR) Biomedical Research Centre at Barts, London, United Kingdom. Drs Petersen, Neubauer, and Piechnik acknowledge the British Heart Foundation, London, United Kingdom, for funding the manual analysis to create a cardiovascular magnetic resonance imaging reference standard for the UK Biobank imaging resource in 5000 CMR scans (PG/14/89/31194). This project was enabled through access to the Medical Research Council eMedLab Medical Bioinformatics infrastructure, supported by the Medical Research Council (MR/L016311/1). Dr Zugwitz acknowledges funding received from the European Society of Cardiology, Sophia Antipolis Cedex, France, in the form of an European Society of Cardiology Training Grant. Dr Neubauer acknowledges support from the Oxford NIHR Biomedical Research Centre and the Oxford British Heart Foundation Centre of Research Excellence. Dr Aung recognizes the NIHR Integrated Academic Training program, which supports his Academic Clinical Lectureship post. Drs McCracken and Neubauer are supported by the Oxford NIHR Biomedical Research Centre. Drs Petersen and Rauseo acknowledge support by the London Medical Imaging and Artificial Intelligence Centre for Value Based Healthcare (AI4VBH), which is funded from the Data to Early Diagnosis and Precision Medicine strand of the government’s Industrial Strategy Challenge Fund, managed and delivered by Innovate UK on behalf of United Kingdom Research and Innovation (UKRI). Dr Nijveldt has received research grants from Philips Volcano and Biotronik. Dr Petersen provides consultancy to Circle Cardiovascular Imaging, Inc. All other authors have reported that they have no relationships relevant to the contents of this paper to disclose.
