# Supplementary material for: Mitral Annular Disjunction Assessed Using CMR Imaging: Insights From the UK Biobank Population Study
Source: JACC Cardiovasc Imaging. 2022 Nov;15(11):1856–66. doi: 10.1016/j.jcmg.2022.07.015 (PMC9640354; doi:10.1016/j.jcmg.2022.07.015)
Supplement: Supplemental Figure 1 and 2 and Tables 1 and 2 [file mmc1.docx]

***Supplementary Table 1: UK Biobank data sources for cardiovascular diseases***

| **Source** | **UKB Field ID** | **Value** | **Description** |
| --- | --- | --- | --- |
| ***Hypertension*** |  |  |  |
| Self-reported illness | 20002 |  | hypertension |
| Medication codes | 6177, 6153 | 2 | Blood pressure medication |
| ICD10 Summary diagnoses | 41270, 41280 | I10 | Essential (primary) hypertension |
| First occurrences | 131286 | Date | Date first diagnosis: Essential (primary) hypertension |
| Diagnosed by doctor | 2966 | Age | Age high blood pressure diagnosed |
| ***Ischemic heart disease*** |  |  |  |
| Self-reported illness | 20002 |  | angina |
|  |  |  | heart attack/myocardial infarction |
| ICD10 Summary diagnoses | 41270, 41280 | I20 | I20 Angina pectoris |
|  |  | I21 | I21 Acute myocardial infarction |
|  |  | I22 | I22 Subsequent myocardial infarction |
|  |  | I23 | I23 Certain current complications following acute myocardial infarction |
|  |  | I24 | I24 Other acute ischemic heart diseases |
|  |  | I25 | I25 Chronic ischemic heart disease |
| First occurrences | 131296 | Date | Date first diagnosis: Angina pectoris |
|  | 131298 | Date | Date first diagnosis: Acute myocardial infarction |
|  | 131300 | Date | Date first diagnosis: Subsequent myocardial infarction |
|  | 131302 | Date | Date first diagnosis: Certain current complications following acute myocardial infarction |
|  | 131304 | Date | Date first diagnosis: Other acute ischemic heart diseases |
|  | 131306 | Date | Date first diagnosis: Chronic ischemic heart disease |
| Diagnosed by doctor | 3627 | Age | Age angina diagnosed |
|  | 3894 | Age | Age heart attack diagnosed |
| Algorithmically-defined | 42000 | Date | 42000 Date of myocardial infarction |
| ***Valvular heart disease*** |  |  |  |
| ICD10 Summary diagnoses | 41270, 41280 | I340 | I34.0 Mitral (valve) insufficiency |
|  |  | I38 | I38 Endocarditis, valve unspecified |
|  |  | I390 | I39.0 Mitral valve disorders in diseases classified elsewhere |
|  |  | I391 | I39.1 Aortic valve disorders in diseases classified elsewhere |
|  |  | I398 | I39.8 Endocarditis, valve unspecified, in diseases classified elsewhere |
| First occurrences | 131330 | Date | Date first diagnosis: Endocarditis, valve unspecified |
|  | 131332 | Date | Date first diagnosis: Endocarditis and heart valve disorders in diseases classified elsewhere |
| ***Non-ischemic cardiomyopathies*** | |  |  |
| Self-reported illness | 20002 |  | cardiomyopathy |
|  |  |  | hypertrophic cardiomyopathy (hcm/hocm) |
| ICD10 Summary diagnoses | 41270, 41280 | I110 | I11.0 Hypertensive heart disease with (congestive) heart failure |
|  |  | I119 | I11.9 Hypertensive heart disease without (congestive) heart failure |
|  |  | I130 | I13.0 Hypertensive heart and renal disease with (congestive) heart failure |
|  |  | I131 | I13.1 Hypertensive heart and renal disease with renal failure |
|  |  | I132 | I13.2 Hypertensive heart and renal disease with both (congestive) heart failure and renal failure |
| ICD10 Summary diagnoses | 41270, 41280 | I139 | I13.9 Hypertensive heart and renal disease, unspecified |
|  |  | I420 | I42.0 Dilated cardiomyopathy |
|  |  | I421 | I42.1 Obstructive hypertrophic cardiomyopathy |
|  |  | I422 | I42.2 Other hypertrophic cardiomyopathy |
|  |  | I425 | I42.5 Other restrictive cardiomyopathy |
|  |  | I428 | I42.8 Other cardiomyopathies |
|  |  | I429 | I42.9 Cardiomyopathy, unspecified |
|  |  | I431 | I43.1 Cardiomyopathy in metabolic diseases |
|  |  | I432 | I43.2 Cardiomyopathy in nutritional diseases |
|  |  | I438 | I43.8 Cardiomyopathy in other diseases classified elsewhere |
| First occurrences | 131288 | Date | Date first diagnosis: Hypertensive heart disease |
|  | 131292 | Date | Date first diagnosis: Hypertensive heart and renal disease |
|  | 131338 | Date | Date first diagnosis: Cardiomyopathy |
|  | 131340 | Date | Date first diagnosis: Cardiomyopathy in diseases classified elsewhere |
| ***Heart failure*** |  |  |  |
| Self-reported illness | 20002 |  | heart failure/pulmonary edema |
| ICD10 Summary diagnoses | 41270, 41280 | I500 | I50.0 Congestive heart failure |
|  |  | I501 | I50.1 Left ventricular failure |
|  |  | I509 | I50.9 Heart failure, unspecified |
| First occurrences | 131354 | Date | Date first diagnosis: Heart failure |
| ***Cardiac arrhythmia*** |  |  |  |
| Self-reported illness | 20002 |  | SVT / supraventricular tachycardia |
|  |  |  | atrial fibrillation |
|  |  |  | atrial flutter |
| ICD10 Summary diagnoses | 41270, 41280 | I441 | I44.1 Atrioventricular block, second degree |
|  |  | I442 | I44.2 Atrioventricular block, complete |
|  |  | I460 | I46.0 Cardiac arrest with successful resuscitation |
|  |  | I461 | I46.1 Sudden cardiac death, so described |
|  |  | I469 | I46.9 Cardiac arrest, unspecified |
|  |  | I470 | I47.0 Re-entry ventricular arrhythmia |
|  |  | I471 | I47.1 Supraventricular tachycardia |
|  |  | I472 | I47.2 Ventricular tachycardia |
|  |  | I479 | I47.9 Paroxysmal tachycardia, unspecified |
|  |  | I480 | I48.0 Paroxysmal atrial fibrillation |
|  |  | I481 | I48.1 Persistent atrial fibrillation |
|  |  | I482 | I48.2 Chronic atrial fibrillation |
|  |  | I483 | I48.3 Typical atrial flutter |
|  |  | I484 | I48.4 Atypical atrial flutter |
|  |  | I489 | I48.9 Atrial fibrillation and atrial flutter, unspecified |
|  |  | I490 | I49.0 Ventricular fibrillation and flutter |
|  |  | I492 | I49.2 Junctional premature depolarization |
|  |  | I493 | I49.3 Ventricular premature depolarization |
|  |  | I494 | I49.4 Other and unspecified premature depolarization |
| First occurrences | 131346 | Date | Date first diagnosis: Cardiac arrest |
|  | 131348 | Date | Date first diagnosis: Paroxysmal tachycardia |
|  | 131350 | Date | Date first diagnosis: Atrial fibrillation and flutter |

***Supplementary Table 2: Intra- and inter-observer variability, intraclass coefficient***

|  | Intraobserver variability  [95 % CI] | Interobserver variability  [95 % CI] |
| --- | --- | --- |
| Anterior | 0.93 [0.89, 0.95] | 0.79 [0.59, 0.88] |
| Anterolateral | 0.93 [0.89, 0.95] | 0.92 [0.89, 0.95] |
| Inferior | 0.94 [0.91, 0.96] | 0.84 [0.77, 0.89] |
| Inferolateral | 0.95 [0.92, 0.96] | 0.72 [0.59, 0.81] |

***Supplementary Figure 1: Case selection flowchart***


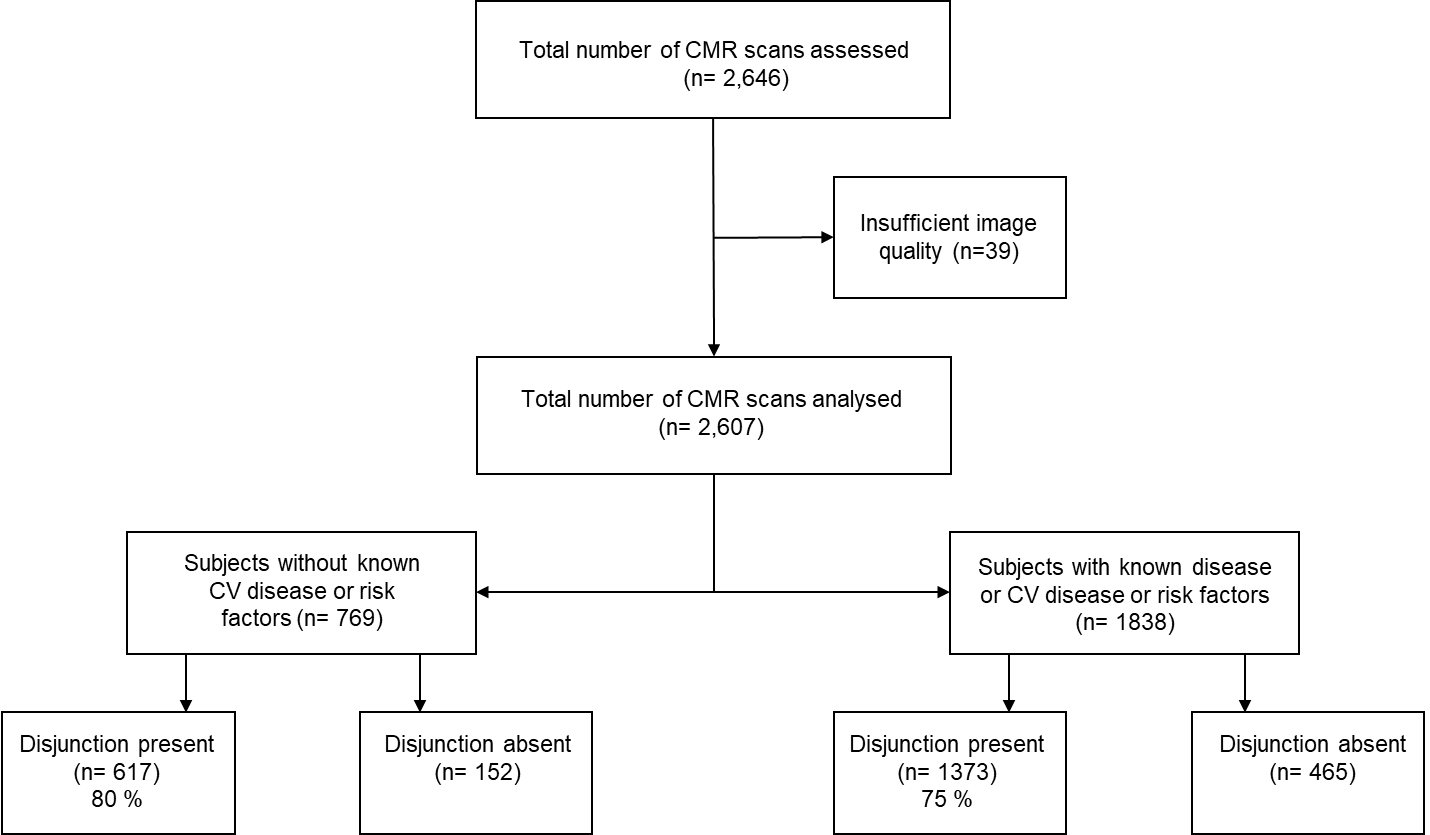


CMR=cardiac magnetic resonance, CV=cardiovascular

***Supplementary Figure 2:***

**
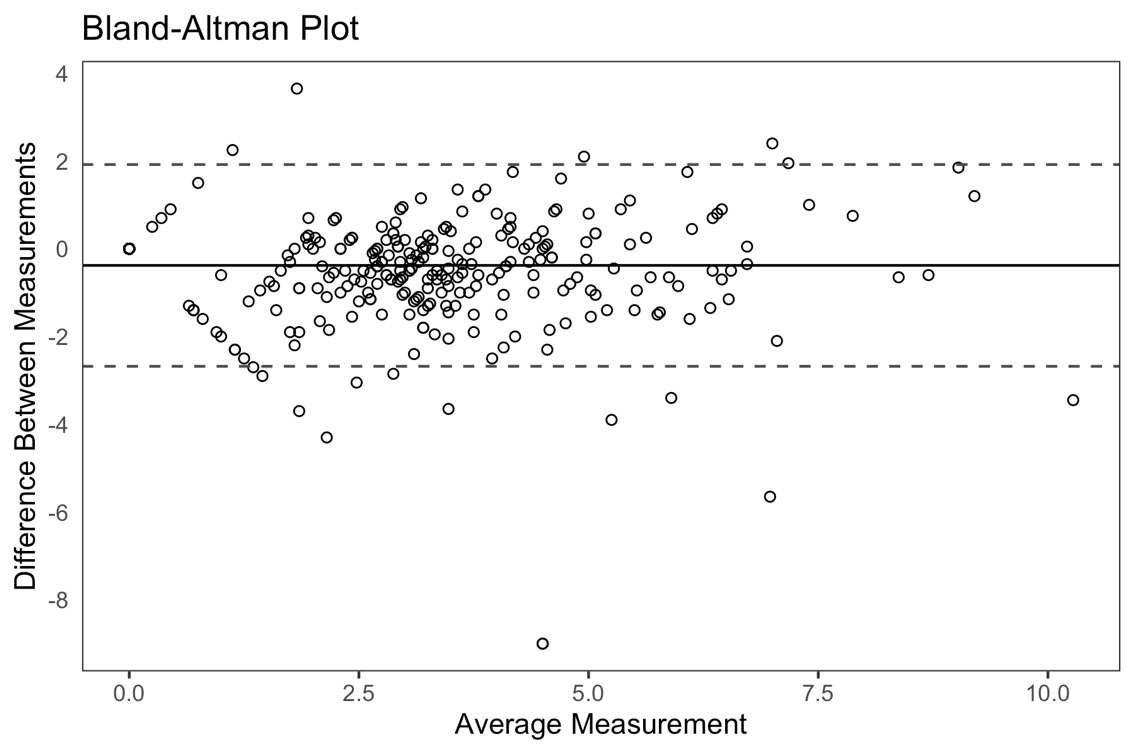
**
